# Inflammatory Bowel Disease and the Environment: More to Come

**DOI:** 10.1093/crocol/otaa048

**Published:** 2020-06-02

**Authors:** Wael El-Matary

**Affiliations:** Section of Pediatric Gastroenterology, Department of Pediatrics, Max Rady College of Medicine, Rady Faculty of Health Sciences and Children’s Hospital Research Institute of Manitoba, Winnipeg, Manitoba, Canada

Inflammatory bowel disease (IBD) is a chronic immune-related disorder that affects gastrointestinal tract. As the disease is non-curable, patients need a lifelong treatment with a typical disease course of remissions and relapses.^1–3^ The etiopathogenesis of IBD is still unclear; however, many identified and non-identified environmental factors play a major role in intestinal dysbiosis with subsequent immune dysregulation in genetically susceptible individuals.^1–4^ Several environmental factors such as nicotine smoking, early exposure to breastfeeding, antibiotics, air pollution, and rurality have been examined. One of the major environmental determinants in IBD pathogenesis is diet.^4,5^ Several dietary factors have been explored and linked to the pathogenesis of IBD such as vitamin D intake.^5–7^ Obesity, on the other hand, has been recognized as a major health problem with a concerning surge in prevalence, especially in Western countries. Obesity is known to be associated with an inflammatory state and its link to IBD has been the subject of ongoing basic and clinical research over the last several years.^8,9^ Obese persons with IBD may have a higher rate of disease relapse, resistance to medical therapy, and the need for IBD-related surgery.^10,11^

In an interesting review, Szilagyi et al^12^ compared rates of obesity, IBD, with the geographic markers of lactase digestion status, average population-weighted national latitude, and national yearly sunshine exposure in 47 countries across the globe. The main findings shown were the global modest to moderate correlations of Crohn’s disease (CD) and ulcerative colitis (UC) incidence with geographic markers. The correlation between obesity and IBD (both CD and UC) was globally moderate, but it was poor for Europe and Asia. As the obesity pandemic is more pronounced in Western countries, it is not clear why the correlation was poor in Europe.

The correlation of CD incidence with lactase non-persistence (LNP) in Europe and the correlation of CD and UC incidence with latitude in Asia remained moderate to strong. When the prevalence data on IBD were assessed, the outcomes with the geographical markers were similar globally but less clear in Europe and Asia, where UC prevalence remains moderately associated with LNP and strongly associated with latitude in Asia. It is difficult to know what this exactly means or the implications of these findings.

While the study is interesting, it is limited by the availability and accuracy of epidemiological data from different countries. The results of the study are dependent on several assumptions and calculations that might be difficult to interpret. The accuracy of these calculations needs to be validated in future studies.

## Notes

Data availability: Data sharing is not applicable to this article, as no new data were created or analyzed in this article.
